# Surprise Packets of Wartime

**Published:** 1917-02-10

**Authors:** 


### THE MATRONS' AND SISTERS' DEPARTMENT.

### SURPRISE PACKETS OF WARTIME.

THE surprise packet of wartime has undoubtedly been the extraordinary development displayed by many women workers in all portions of the nursing field, and especially when engaged on foreign service. Some of the quietest of matrons who had attained a reputation and marked success in the management of voluntary hospitals previous to the outbreak of war, have shown extraordinary development by the exhibition of untiring energy and the acceptance of the hardest conditions, without a murmur or any apparent loss of health or keenness for their work. We have known some of the higher type of women workers who have tried to adapt themselves to new fields of labour, which they had shown conspicuous evidence of a capacity to cultivate, that when transferred from institutional life to a position of independence, failed to fulfil under the altered conditions the initial promise. Again, there are a very large number of nurses working during the war in connection with so-called hospitals, some of them large ones situated even in London, where sometimes the health conditions and accommodation for the nursing staff, sometimes the food, its quality or quantity, or both, sometimes the absence of adequate discipline and the presence of sound administrative management, have made the work of many a sister and nurse unnecessarily arduous, or calculated to diminish her energies and affect her health. Only very occasionally does a nurse complain, and the reason has often been that those responsible for grave defects, as we regard them, and for the unnecessary and cruel conditions, have covered up their own faults by enforcing a false standard of so-called loyalty on the Nurses, and have so closed the mouths of the staff, *because it is wartime*.

The war is approaching the termination of its third year, and we feel that in a few notorious cases, unless the conditions under which the nurses are housed, have to work and are fed, are changed and brought up to a proper standard, by the completion of the third year of war, an inquiry into those conditions must be continuously insisted upon until Parliament acts, and those responsible are brought to the bar of public opinion. Anyone with a knowledge of the facts, who has had experience of hospital administration, knows that in nearly every one of the cases we have in mind the fault lies with the initial mistake of appointing to the higher offices, persons who, however successful in smaller undertakings, do not possess the qualities, character, and ability efficiently to administer a great hospital or organisation in all its branches.

Looking at these questions from the point of view of the devoted sister or trained nurse we can sympathise with them in bearing inhygienic conditions, rough, inadequate, or badly cooked food, unwarmed rooms, the absence of adequate opportunities for rest, hours of duty to excess, and many other things which ought not to be, and *need not be if the administrative Heads of the faulty institutions were up to their work.* The truth is they have not a standard for work worthy of those employed in attendance on the sick, during a great war, like that we are engaged in at present. Nurses may be so obsessed by the desire to help the wounded, to do their part for the country and the nation during the war, and generally to bear and suffer in the cause of the nation, that we have had instances brought to our notice on several occasions, one only within the past fortnight, where the friends or parents of nurses engaged in some of these so-called war, or military hospitals, have made it their business to take every opportunity to feed the member of their family or their friend so engaged. It is remarkable that the breakdown in health has not created an outcry, the reason being, probably, that nurses engage themselves for a period, and that they manage by great self-denial and control to continue on the war work until their time expires, when they go away to recruit. It passes our comprehension how the authorities of some of these hospitals can see these things going on, as they must do in regard to a large staff of nurses, and not be stirred to the withers to bring about changes which will make the continuance of such evils and cruelties and mismanagement impossible.

Six months ago we called attention to a notorious example of the abuses here referred to. We then said that a state of war, and such a war as the present one, which was forced upon the nation all unprepared, and forced insistently, necessarily created huge difficulties at the outset, not the least of which was to find some one everywhere to be doing something in the way of a start. . This fact necessitated a blind eye on the part of those who were aiming at improvement and higher efficiency in the first year of the war. We are now, however, approaching the expiration of the third year of war. Time and circumstances have demonstrated where there are leaks, in those portions of the hospital system which are directly associated with the fighting forces. It seems to us essential that those leaks should be stopped now, and efficiency substituted everywhere. Since that was written improvements have taken place in various directions, but there are still matters that ought not longer to continue as they are. Indeed, the sooner they are grappled with and ended the better it will be for the reputation of everybody concerned.

